# CREATE Wellness: A multi-component behavioral intervention for patients not responding to traditional Cardiovascular disease management

**DOI:** 10.1016/j.conctc.2017.10.001

**Published:** 2017-10-04

**Authors:** Chris Miller-Rosales, Stacy A. Sterling, Sabrina B. Wood, Thekla Ross, Mojdeh Makki, Cindy Zamudio, Irene M. Kane, Megan C. Richardson, Claudia Samayoa, Nancy Charvat-Aguilar, Wendy Y. Lu, Michelle Vo, Kimberly Whelan, Connie S. Uratsu, Richard W. Grant

**Affiliations:** a University of California at Berkeley, Berkeley, CA, USA; b Division of Research, Kaiser Permanente, Oakland, CA, USA

**Keywords:** Patient-centered, Intervention research, Stakeholder engagement research

## Abstract

**Background/Aims:**

Cardiovascular disease (CVD) is the leading cause of death in the US. Many patients do not benefit from traditional disease management approaches to CVD risk reduction. Here we describe the rationale, development, and implementation of a multi-component behavioral intervention targeting patients who have persistently not met goals of CVD risk factor control.

**Methods:**

Informed by published evidence, relevant theoretical frameworks, stakeholder advice, and patient input, we developed a group-based intervention (Changing Results: Engage and Activate to Enhance Wellness; “CREATE Wellness”) to address the complex needs of patients with elevated or unmeasured CVD-related risk factors. We are testing this intervention in a randomized trial among patients with persistent (i.e > 2 years) sub-optimal risk factor control despite being enrolled in an advanced and highly successful CVD disease management program.

**Results:**

The CREATE Wellness intervention is designed as a 3 session, group-based intervention combining proven elements of patient activation, health system engagement skills training, shared decision making, care planning, and identification of lifestyle change barriers. Our key learnings in designing the intervention included the value of multi-level stakeholder input and the importance of pragmatic skills training to address barriers to care.

**Conclusions:**

The CREATE Wellness intervention represents an evidence-based, patient-centered approach for patients not responding to traditional disease management. The trial is currently underway at three medical facilities within Kaiser Permanente Northern California and next steps include an evaluation of efficacy, adaptation for non-English speaking patient populations, and modification of the curriculum for web- or phone-based versions.

**ClinicalTrials.gov Identifier:**

NCT02302612.

## 1 Introduction

Cardiovascular disease (CVD) represents an enormous burden of disease in the US, accounting for one-third of U.S. deaths per year [1]. With improved survival rates, there are now over 80 million Americans living with CVD [2]. Diagnosis and treatment of modifiable CVD risk factors such as hypertension and hyperlipidemia represent one of the major successes in the effort to reduce CVD-related morbidity and mortality in the US [3]. Despite promising trends, however, the majority of patients with CVD still do not reach evidence-based risk reduction goals [4]. New primary care delivery strategies are therefore needed to ensure more effective implementation of evidence-based CVD risk reduction therapies.

Disease management programs have been widely implemented for common conditions such as hypertension, diabetes, and heart failure [5], [6], [7]. These programs generally include tools such as population-level screening, laboratory result monitoring, telephone-based outreach by nurses, and disease-specific medication titration protocols to achieve evidence-based and disease-specific clinical goals. While often successful for single conditions or inter-related risk factors, traditional disease management has often not been sufficient for patients with health issues and care barriers that extend beyond a narrow disease-specific focus [8].

Management of CVD risk factors is complicated by the increasingly high prevalence of concurrent comorbid diagnoses [9]. Among Medicare beneficiaries, for example, 40% suffer from at least 3 chronic comorbid conditions and over 20% have more than 4 conditions [10]. Multiple comorbidity does not necessarily preclude effective CVD risk reduction, but within the CVD patient population there exists a subset of patients with characteristics (e.g. lower patient activation and engagement with health care, lower levels of motivation) and/or mental health comorbidities (e.g. depression, anxiety, alcohol misuse) that may present modifiable barriers to effective care.

Further progress in CVD risk factor control in these patients with complex conditions will require new approaches to care. We hypothesized that a coordinated behavioral approach designed to address common underlying barriers and to provide patients with self-management skills and techniques applicable across multiple different chronic diseases would result in better CVD outcomes compared to usual care. In this paper, we describe the rationale, development, and implementation of an evidence-based, patient-centered behavioral intervention targeting patients within a CVD disease management program who have persistently not met goals of CVD risk factor control.

## 2 Material and methods

### 2.1 Setting

Kaiser Permanente Northern California (KPNC) is an integrated health care delivery system serving more than 4.1 million members. The membership is demographically and socioeconomically diverse [11] and includes more than 350,000 members with CVD risk factors (e.g., hypertension, elevated lipids, poor glycemic control). These patients are automatically enrolled in the successful Preventing Heart Attacks and Strokes Everyday (PHASE) program, a robust, population-based CVD management program implemented in 2005. Key features of PHASE include a continuously updated CVD registry, provider performance feedback, system-wide efficiencies, population management, and evidence-based practice guidelines. Although approach has produced impressive population-level benefits [12], approximately 15% of patients remain persistently uncontrolled over time and are the focus of this intervention.

### 2.2 Conceptual models for intervention

We hypothesized that patients failing traditional disease management may require a multi-component intervention focused on patient self-care knowledge, skills, and confidence, rather than additional disease-specific education or treatment. We therefore sought to create an integrated program designed to activate core patient self-management skills and to introduce self-management strategies for overcoming barriers to care. Based on a scoping review of the literature, we identified four areas of opportunity for supporting patients who are failing traditional disease management. These domains were identified based on evidence of clinical impact among complex patients. Below we describe each of these domains and provide key citations that support their relevance to our intervention development goals.

*Increasing Patient Activation Levels*: Patient activation is defined as understanding one's role in the care process and having the knowledge, skill and confidence to manage one's health and health care [13]. As patients become more “activated,” they build their capacity to take greater responsibility for achieving health-related goals [14], [15], [16]. Patient activation can be reliably measured across adult age ranges and racial/ethnic groups using the Patient Activation Measure (PAM), a 13-item questionnaire that correlates with health outcomes and self-management behaviors including medication adherence [17]. Patients with lower PAM scores are more likely to miss appointments, report lower confidence and knowledge, and apply fewer strategies for problem solving [18]. Interventions based on shared decision making, group learning and motivational interviewing (MI) have successfully raised PAM scores [19], [20], and randomized trials have shown that interventions designed specifically to increase PAM scores result in better clinical outcomes, such as increased minutes of walking among older adults [21] and decreased heart failure hospitalization rates [22].

*Improving Patient Engagement/System Navigation Skills:* Patient engagement is defined as the actions individuals must take to obtain the greatest benefit from the health care services available to them [23]. Because “activated” patients must still find ways to successfully navigate an often convoluted medical system, pragmatic skills training is an essential component to overcoming barriers to care. Prior work has demonstrated the value of training in system navigation (e.g. use of electronic patient portals to communicate with providers) [24] and in problem-solving (e.g. disease-specific education about how to manage home data, symptoms, and side effects; and more general training in how to communicate with providers and to prioritize for decision-making) [25], [26], [27], [28].

*Screening for Behavioral Risk:* Among patients with poor disease control, factors such as risky alcohol use, depressed or anxious mood, sedentary lifestyle and poor diet are prevalent, detrimental, and yet often clinically under-recognized [29]. Because of the challenges to effective health care navigation posed by co-occurring mental health and substance use problems, interventions to activate and engage patients with complex conditions should include activities designed to help identify and address these concerns. Prior work has demonstrated the feasibility and effectiveness of comprehensive screening for clinically unrecognized and untreated behaviors that can undermine attempts to manage complex clinical conditions [30]. Moreover, while more severe behavioral health disorders (e.g. chemical dependency, major depression) identified through screening will require specialty referral, mild or moderate mental health symptoms and substance use can often be effectively addressed through brief group counseling and motivational interviewing [31], [32], [33].

*Care Planning:* One challenge of designing interventions for patients with complex conditions is that while there are clear commonalities in the experience of chronic disease self-management that transcend specific diagnoses, each patient nonetheless presents a unique history of barriers, strengths, preferences, knowledge, skills and goals. Priority-setting through shared decision making can make subsequent clinical encounters more efficient and productive by focusing clinical efforts on the most amenable targets [34], [35], [36]. For patients with complex medical comorbidities, a key benefit of this approach is the ability to collaboratively establish a care plan suited to the patient's level of self-management activation [37], [38]. Matching intervention activities to the patient's level of activation can have lasting impacts on accomplishing and maintaining overall health goals. For example, creating a care plan which builds on the patient's own skills, strengths and preferences can translate the general benefits of interventions into specific actionable goals that each patient can share with his or her care team.

**Fig. 1:**
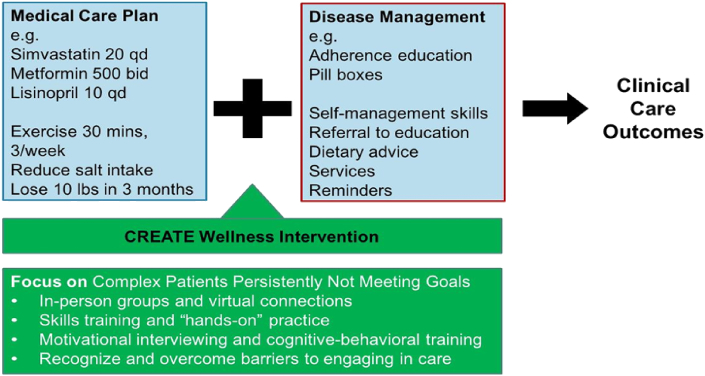
Conceptual model for how the CREATE Wellness intervention supports the Medical Care Plan developed by the patient's physician and the existing Disease Management process to help achieve improved clinical care outcomes. Patients randomized to usual care continue with physician-defined Medical Care Plan and Disease Management, whereas patients randomized to the CREATE Wellness intervention participate in three group-based sessions over six weeks designed to support traditional disease management by increasing patient activation, engagement, and pragmatic self-care skills.

We created an intervention program that combines elements of these four proven, evidence-based domains. The resulting intervention is designed to augment rather than replace the existing disease management program (Fig. 1). To date there have been no published randomized trials of patient-centered interventions that integrate all four of these components into an intervention for patients with complex conditions not meeting care goals.

### 2.3 Study design considerations

We chose a pragmatic, parallel group, randomized controlled trial design to test our intervention. In keeping with our pragmatic framework, patients randomized to control arm will continue to receive usual care, including the established PHASE disease management program, while intervention patients have the addition of three group based sessions. We randomize at the patient level, and we will make willingness to participate an eligibility criterion to avoid undermining the power for the intervention to show efficacy due to high drop-out or lack of attendance.

### 2.4 Choice of study outcomes

**Fig. 2:**
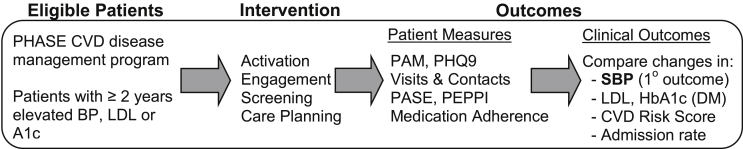
Conceptual Framework showing the flow from enrolling eligible patients, components of the CREATE Wellness intervention, patient-oriented intermediate measures, and downstream clinical outcomes. PAM = Patient Activation Measure, PHQ-9 = Patient Health Questionnaire, PASE = Physical Activity Scale for the Elderly, PEPPI = Perceived Efficacy in Patient-Physician Interactions Questionnaire, SBP = Systolic Blood Pressure, LDL = Low Density Lipoprotein Cholesterol; CVD = Cardiovascular Disease.

Fig. 2 presents a conceptual diagram of hypothesized patient-oriented intermediate outcomes and downstream clinical outcomes. We will collect data through computer-based surveys, follow-up interviews, and via the electronic health record. Although reducing CVD-related mortality is the ultimate goal for interventions to improve CVD risk management, we rely on proven surrogate measures (i.e. improved blood pressure, cholesterol, and glycemic control) which are prevalent and have potential to change during the 12-month study follow-up period.

*Survey Data*: At baseline, following recruitment, study participants will complete a computer-based interview at a private place in the primary care clinic. Follow-up telephone interviews will be conducted at 3 months by research staff. Assessments at both time points will include the 13-item Patient Activation Measure (PAM), [39] 5-item Perceived Efficacy in Patient-Physician Interactions Questionnaire (PEPPI), [40] 9-item Patient Health Questionnaire for depression (PHQ-9), [41] 7-item Generalized Anxiety Disorder survey (GAD-7), [42] Physical Activity Scale for the Elderly (PASE, 12-item), [43] the 5-item EuroQol-5D for quality of life, and Alcohol Use Disorders Identification Test (AUDIT, 10-item) [44].

*Clinical Data*: We will assess changes in CVD risk factor levels over time in the two study arms. We will use data collected as part of clinical care rather than by the study team for the following reasons: 1) Rates of missing data for KPNC disease management patients are extremely low, 2) This strategy underscores the pragmatic and generalizable nature of our intervention, and 3) By not directly collecting clinical data (e.g. via blood draws), research staff can conduct surveys by phone, thereby increasing convenience for participants and reducing missing survey data and costs. For medication adherence, we will use pharmacy dispensing data to calculate Proportion of Days Covered (PDC). We will create aggregate PDCs for CVD-related and non-CVD-related medication adherence. Assessing the impact of the intervention on non-CVD-related medication PDC will provide an estimate of the “cross-over” impact of the intervention on patients' other chronic conditions.

### 2.5 Translating the intervention concept into an intervention program

Developing a multifaceted behavioral intervention requires engagement with a wide range of patient and clinical stakeholders. Grounded in our conceptual framework, we elicited feedback from key stakeholder groups at several stages during the creation and implementation of our program. Stakeholders included patients, clinical health educators, health system leaders and executives, health information technology specialists, primary care and geriatric providers, and population health managers.

We used a multi-pronged, iterative development strategy, including involving key leaders at the local and national level from the start. In times of multiple competing priorities, we found that it was essential to garner interest at the system level in order to demonstrate the direct value and impact of such an intervention [45]. Four stakeholder meetings with health system leaders were conducted as interactive presentations at each stage of intervention development. Participants included national program executives from the medical group, directors of health education and internal quality consulting, and clinical leaders in CVD disease management and behavioral health. Questions addressed during these meetings included: Which patients should we choose (eligibility criteria)? What skills development would be most important for these patients (content development)? and, How should this content be delivered (implementation)?

Patient focus groups were used to identify areas of greatest interest and concern from the patient perspective. Focus group participants were identified using the same eligibility criteria planned for the clinical trial. Specifically, we identified English-speaking adults with >2 years of uncontrolled and/or unmeasured cardiovascular risk factors who were enrolled in the PHASE disease management program. After approval from their PCP, we contacted participants and obtained their informed consent to participate in 90-min focus groups held at the local clinical center. We conducted 8 focus groups over time. Initial sessions focused on the overarching question: “What is keeping you from meeting your health care goals and getting the help you need?” As the intervention structure and content was developed, we elicited comment and feedback from participants to guide further modifications. The focus group discussion guides included a series of overarching, open-ended questions, followed by more specific, clarifying questions. For each major question, prompts reminded the moderator of salient discussion topics and helped stimulate discussion. For all stakeholder and focus group sessions, 3–4 research team members took active field notes which were discussed during follow-up research meetings. Focus groups were also recorded to capture a complete record of what took place, and the recordings transcribed. Two members of the investigative team independently summarized each transcript.

### 2.6 Sample size considerations and analytic plan

All primary analyses will be intention-to-treat. We will carry forward missing data from baseline and also use multiple imputation and conduct sensitivity analyses to assess the possible impact of missing data. For continuous measures, we will use univariate analyses to obtain descriptive statistics and check for normality. We will use t-tests and analysis of variance (ANOVA) techniques to assess effects of covariates (e.g., gender, age) on continuous outcomes (e.g., SBP, PAM scores). For categorical variables, we will calculate frequency distributions and bivariate tables (e.g. % improved PAM by study arm), and then use bivariate odds ratios and chi-square tests to evaluate differences in proportions by study arm. These results will be used to guide multivariate regression analyses. For continuous clinical (e.g. SBP, LDL, FRS, MPR) and survey outcomes (e.g. PAM, PHQ-9, GAD-7 scores), we will first construct multi-level linear regression models, adjusting for clustering by physician and practice as random effects, with intervention (yes/no) as the main independent variable. We will use the mean of available measures in the 12 month period after intervention completion for SBP, LDL, A1c, and FRS. In a secondary analysis, we will also dichotomize outcomes based on standard thresholds and use similar logistic regression models to calculate odds of control (for SBP, LDL, A1c) and adherence (>80% MPR). We will use hierarchical models that include a fixed effect for the intervention and random effects for PCP and practice to account for the intra-class correlations (ICC) across patients within providers and within practices.

*Power Analysis and Sample Size*: We present here our calculations used to determine study sample size for detecting differences between study arms in our primary outcome of systolic blood pressure (SBP). Although we randomize at the patient level, measures of patients cared for by the same PCP and PCPs within the same practice may be slightly correlated. To account for these correlations, our power calculations include an intra-class correlation (ICC) of ≤1% based on prior work. We also conservatively assume a 15% attrition rate and 95% SBP result availability. With these assumptions, a plan to enroll 576 study subjects (288 per arm) will result in nominal sample of 466 subjects with SBP values after attrition, and an effective sample of 390 after accounting for ICC. This sample provides 90% power to detect a 3.45 mm Hg difference between study arms assuming standard deviation (SD) of 10.5 mm Hg. With this sample size, we have 90% power to detect an 11.8 mg/L difference in LDL Cholesterol (secondary clinical outcome) and 80% power to detect a 4.6 difference in PAM score (secondary survey outcome).

## 3 Results

### 3.1 Stakeholder input

**Fig. 3:**
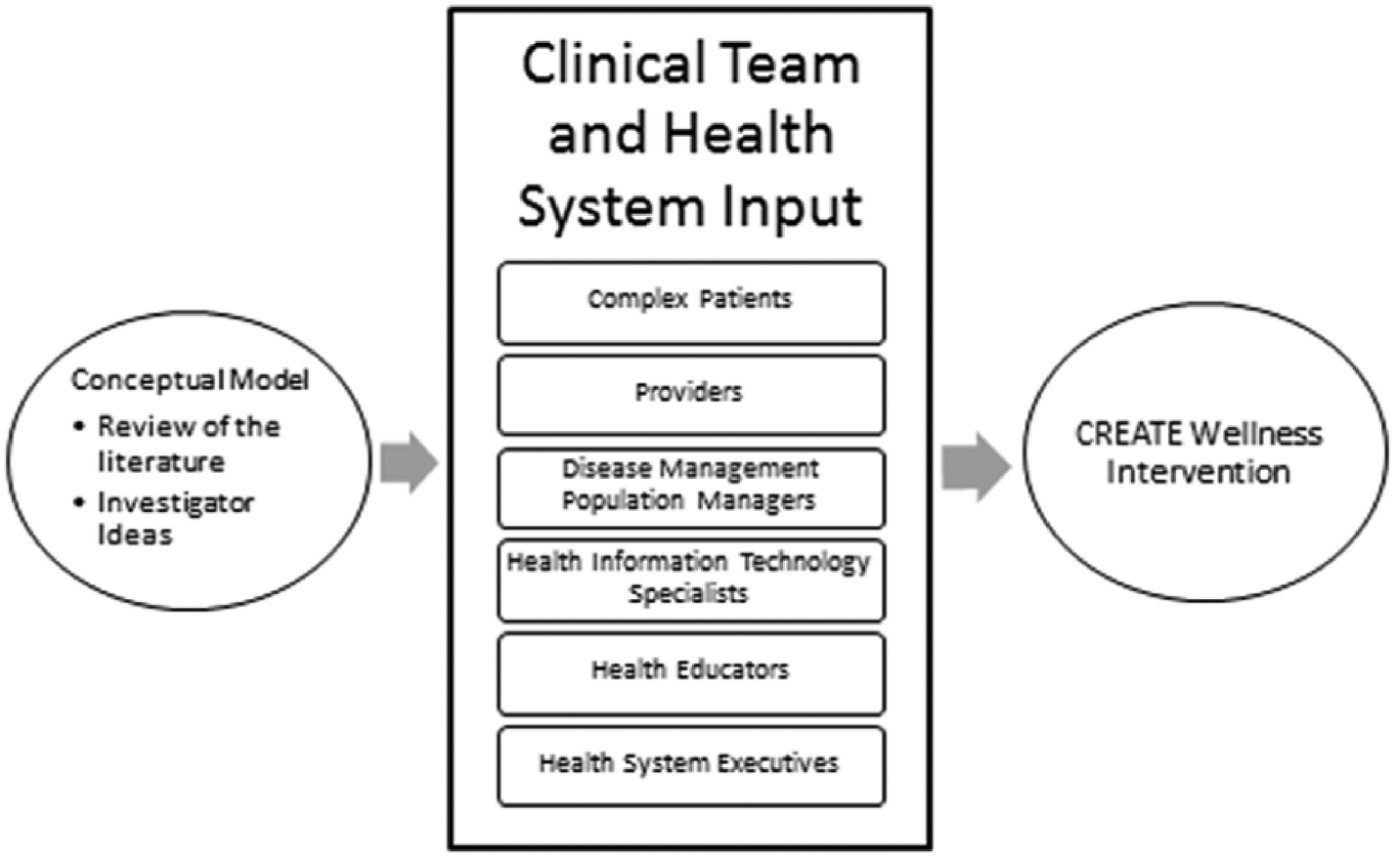
Illustration of how initial intervention plans (based on review of literature and investigator ideas) were modified by input from multiple stakeholders to design to final CREATE Wellness intervention.

Stakeholder engagement resulted in multiple key modifications and changes to our initial intervention plans (Fig. 3). From our health system leaders and clinicians, we learned how to refine the subset of patients we should focus on within the disease management program, how to best position this intervention within ongoing efforts in the care system, and how to optimize the role of health information technology and innovation. Stakeholder “buy-in” was crucial to the study because it stimulated interest in the project, provided critical input on intervention content, facilitated clinical implementation, and increased leaders' investment in the project. Some examples of study adaptations based on stakeholder engagement:

1) *Patient Selection*: We approached stakeholders for their advice on which subset of patients within the disease management program we should pursue. Initially we considered using prioritization algorithms to target patients with the most need, but stakeholders did not see value in trying to predict which group would benefit most based on clinical characteristics, and we therefore decided to pursue a more general population of patients persistently not meeting care goals.
2) *Pragmatic Application*: It was clear from stakeholder feedback that navigation of the web-based patient portal would be a fundamental aspect of the intervention patient experience. Clinicians and care managers are able to see if a patient has an open health portal account, but are unable to see if it is actively used. We also received patient feedback that they may be registered for the site but have never been able to sign in. A critical component of implementation was gaining the necessary institutional permissions for interventionists to change participants' passwords during the group sessions, thereby reducing a key barrier effective system navigation.

From our patient stakeholders, we learned how to tailor evidence-based concepts to fit the needs and preferences of patients. Once we had a “working prototype” of the proposed intervention, we conducted mock sessions with patient stakeholders to refine our presentation, identify areas for increased attention, and help train our interventionists in delivering the curriculum.

### 3.2 The CREATE Wellness intervention

Based on our conceptual framework and stakeholder input, we created an intervention entitled “Changing Results: Engaging and Activating To Enhance Wellness” (CREATE Wellness). We designed a 3-session group-based curriculum with supplemental one-on-one contacts, delivered over 6 weeks.

*Session 1: Navigating the System to Meet My Needs -* The first session includes an overview of the program, group introductions, and begins the process of patient engagement. The interventionist engages the patients in a collaborative review of the Kaiser Permanente online health portal and troubleshoots individual barriers to accessing care, including re-setting account passwords. Patients develop the knowledge and skill to navigate the online health resources, including emailing their doctor. The interventionist also assists patients with smart phones to download the Kaiser Permanente health portal applications.

To support behavior change, our interventionists undergo Kaiser Permanente motivational interview training (MI) and apply these techniques (e.g., rolling with resistance, empathy, summarizing, reflective listening and developing discrepancies between health goals and current behaviors) to aid study participants in setting realistic and identifiable overall health goals [46]. In the CREATE Wellness sessions, patients are encouraged to gain the necessary knowledge and skill to be more confident practicing behaviors that enhance their overall wellbeing. Further, patients are taught to normalize failures and setbacks. This session is intended to help participants learn about available healthy lifestyle tools and to access health resources that help them cope with the challenges of managing chronic conditions and overall health now and in the future.

*Session 2: Engage in Wellness -* In the second session, patients have an opportunity to share with each other what is working and not working with regard to their health goals and maintaining overall healthy lifestyle behaviors (e.g. nutrition, exercise, taking medications as prescribed). The previous session encouraged the use of health portals, and in this session patients discuss their experience being active online members since the last meeting.

Medications are a key component of care for patients with complex diseases, and stakeholder input indicated that we needed to incorporate discussion of medication adherence. Through open dialogue, patients share their own barriers to effective medication management and find strategies to address those barriers including disbelief in the efficacy of the medications. In this session the interventionist spends time reflecting on member's experiences, building empathy and highlighting change talk. The group explores why readiness plays a crucial role in maintaining behavior over time.

*Session 3: Establish a Plan: Preparing, Communicating, and Participating In Your Care -* During the third session, patients have an opportunity to identify their own concerns that they would like to discuss with their providers at an upcoming visit. Patients fill out a care plan to prepare for their appointment that includes health goals and personal preferences. During this session patients practice discussing their medical concerns and personal experiences in a structured format that links to their overall health goals. Patients are encouraged to send an outline to their PCP via the electronic medical record so the doctor will be prepared to address their goals and questions during the visit. Further, participants use role play to practice how they may effectively communicate with various members of the care team to address their health care goals. When discussing effective communication, the interventionist addresses cultural norms and social barriers that impede assertive communication.

*Between visit contacts -* The interventionist uses motivational enhancement strategies to engage patients at an individual level between groups. Each participant is contacted between group sessions via telephone or secure electronic message. These between session intractions are intended to continue building member confidence through active practice and also as an opportunity to focus on individual care goals.

### 3.3 Training and fidelity plans

We are closely following the recommendations of the Treatment Fidelity Workgroup of the National Institutes of Health Behavior Change Consortium [47] regarding the fidelity of design, provider training, delivery, receipt, and enactment of behavioral interventions. To address the domain of design, we will ensure that all intervention patients attend 3 curriculum sessions by providing make-up opportunities. We will keep training in MI and curriculum content consistent for all interventionists and have a “refresher” training session after 1 year. We also developed a training manual that summarizes all aspects of the intervention, including the conceptual model, session outlines, handouts, and suggested reading. To ensure fidelity of delivery, intervention sessions are observed by a trained research assistant and coded over 9 elements of the curriculum in a fidelity checklist. To measure enactment we will document whether intervention patients bring their Care Plan to a primary care visit. This treatment fidelity assessment framework will also allow us to examine contextual factors that affect implementation and adoption (e.g., necessary personnel, training, feasibility, staff and/or leadership acceptance).

## 4 Discussion

Cardiovascular disease (CVD) is the leading cause of death in the US. However, the majority of patients with CVD still do not reach evidence-based risk reduction goals [48]. Disease management programs have emerged as a powerful tool to help populations of patients reduce their CVD risk [49]. While often successful for many patients, these traditional disease management programs have not had the same impact on patients with complex comorbidities or other barriers to effective care [8]. To help address this care gap, we designed and implemented a behavioral intervention designed to activate and engage patients using a patient-centered rather than disease-focused approach.

We integrated multiple evidence-based concepts from published literature to develop the CREATE Wellness program, an innovative multi-modal intervention designed to address the unique needs of a complex patient population not responding to traditional disease management strategies. A robust and iterative process of stakeholder feedback informed both the fundamental components of our intervention and also the scope of our target population, resulting in an intervention that includes pragmatic skills training and is delivered with motivational interviewing and behavior change techniques.

Several potential limitations of our study should be noted. Focus groups may be influenced by how the interviewer frames the issues and guides the discussion, and our chosen focus group participants may not be entirely representative of the larger population. We accounted for the first issue by using more than one interviewer, having an interview guide, and transcribing then reviewing the discussions. We selected focus group participants using the same criteria as planned for the clinical trial and used multiple sessions to account for variability.

Next steps will include an evaluation of the efficacy of the CREATE Wellness intervention in a randomized controlled trial with both patient-oriented and clinical outcomes compared to usual care that includes the traditional CVD disease management program that is already in place. If this intervention proves successful, subsequent work will include modifying the curriculum for future delivery in other formats, including less-resource intensive interactive web- or phone-based sessions. Given the important influence of culture and language on patient engagement with health care, future work will also include adaptation of our intervention for non-English speakers from different cultures.

The persistent inability to achieve comprehensive CVD risk reduction for all patients represents an important limitation of our current care delivery system. While disease management programs represent an advance over traditional visit-based primary care, these disease-focused programs may not be optimal for the growing population of CVD patients with multiple other chronic conditions. By providing patients with the tools to understand their health issues and to find the right resources to fix them, the CREATE Wellness intervention seeks to empower patients as the focal agent for catalyzing change along the entire pathway to clinical outcomes. If found to be successful, this approach to developing and implementing a patient-focused intervention can be generalized to other chronic and complex conditions.

## Funding

This work was supported by National Heart, Lung, and Blood Institute (Grant Number HL117939).
